# Supplementary material for: A 25-Year Retrospective on Bavaria’s Newborn Screening Programme: Achievements, Challenges and Long-Term Follow-Up
Source: Int J Neonatal Screen. 2025 Dec 13;11(4):114. doi: 10.3390/ijns11040114 (PMC12733661; doi:10.3390/ijns11040114)
Supplement: Supplementary file 1 [file IJNS-11-00114-s001.zip › ijns-3904159-supplementary.pdf]

Supplementary Materials

Suppl. Table S1. Positive predictive values (PPV) from 25 years of the Bavarian Newborn Screening programme (1999-2023; N=2,854,190), stratified by strength of indication for the condition as given by the NBS laboratory.

	**Total NBS results**			**Borderline NBS results[1]**			**NBS results with strong indication for the condition[1]**		
	**Recall**	**Cases[2]**	**PPV [%]**	**Recall**	**Cases**	**PPV [%]**	**Recall**	**Cases**	**PPV [%]**
Hypothyroidism	2,100	890	42.38	1,321	240	18.17	779	649	83.31
CAH	11,881	205	1.73	11,552	44	0.38	329	161	48.94
Biotinidase deficiency	599	53	8.85	551	19	3.45	48	34	70.83
Classic galactosemia	1,410	43	3.05	1,332	8	0.60	78	35	44.87
PKU / mild HPA	1,076	575	53.44	612	184	30.07	464	391	84.27
MSUD	62	11	17.74	41	4	9.76	21	7	33.33
MCADD	441	297	67.35	172	66	38.37	269	231	85.87
LCHADD	116	14	12.07	97	6	6.19	19	9	47.37
VLCADD	191	29	15.18	133	12	9.02	58	17	29.31
Carnitine cycle defects	98	8	8.16	85	4	4.71	13	4	30.77
GA I	176	22	12.50	136	4	2.94	40	18	45.00
IVA	491	25	5.09	399	5	1.25	92	20	21.74
Tyrosinemia type 1	33	3	9.09	30	2	6.67	3	1	33.33
SCID	157	8	5.10	145	4	2.76	12	4	33.33
SCD[1]	77	41	53.95						
SMA[1]	35	33	94.29						
CF[1]	971	205	21.11						
**Total**	**19,914**	**2,462[2]**	**12.36**	**16,606**	**602**	**3.63**	**2,225**	**1,581**	**71.06**

[1] For SCD, SMA, and CF the strength of indication is not graded, [2] excluding 40 newborns not identified through NBS (false negative, n=35; NBS declined, n=5)

Abbreviations:: CAH, congenital adrenal hyperplasia; CF, cystic fibrosis (mucoviscidosis); GA I, glutaric acidemia type I; HPA, hyperphenylalaninemia; IVA, isovaleric acidemia; LCHADD, long-chain 3-hydroxyacyl-CoA dehydrogenase deficiency; MCADD, medium-chain acyl-CoA dehydrogenase deficiency; MSUD, maple syrup urine disease; PKU, phenylketonuria; PPV, positive predictive value; SCD, sickle cell disease; SCID, severe combined immunodeficiency; SMA, 5q spinal muscular atrophy; VLCADD, very-long-chain acyl-CoA dehydrogenase deficiency.

Suppl. Table S2. Details on confirmatory diagnostics in the Bavarian Newborn Screening long-term programme over 25 years (1999-2023).

**Condition**	**Total (n)**	**Confirmation in specialised centre of expertise (n= 2,500)**		**Age at initiation of care or therapy (n=2,500)**					
		**Yes**	**No**	**≤ 7 days**	**8-10 days**	**11-14 days**	**14-28 days**	**> 28 days**	**N/A[1]**
Hypothyroidism[2]	899	408	491	644	90	37	30	20	78
CAH[3]	217	127	90	146	29	14	10	8	10
Biotinidase deficiency	54	48	6	9	15	7	8	10	5
Classic galactosemia	44	39	5	39	0	0	2	1	2
PKU	257	244	13	176	53	8	5	5	10
Mild HPA	319	286	33	76	63	53	72	38	17
MSUD	12	12	0	10	1	0	0	0	1
MCADD	296	259	37	150	62	29	24	18	13
VLCADD	31	30	1	21	4	1	3	0	2
LCHADD	15	15	0	10	0	2	0	1	2
Carnitine cycle defects	8	7	1	6	2	0	0	0	0
GA I	24	24	0	15	4	1	1	1	2
IVA	25	21	4	14	4	1	3	2	1
Tyrosinemia type I	3	3	0	3	0	0	0	0	0
SCID	8	7	1	3	2	1	2	0	0
SCD	41	38	3	3	2	6	18	12	0
SMA	33	30	3	19	9	3	2	0	0
CF	214	207	7	9	0	13	125	58	9
**Total**	**2,500**	**1,805**	**695**	**1,353**	**340**	**176**	**305**	**174**	**152**

[1] N/A= not available: NBS false-negative, declined, or conducted in premature infant; [2] One child with an additional diagnosis of HPA; [3] One child with an additional diagnosis of MCADD

Abbreviations: CAH, congenital adrenal hyperplasia; CF, cystic fibrosis (mucoviscidosis); GA I, glutaric acidemia type I; HPA, hyperphenylalaninemia; IVA, isovaleric acidemia; LCHADD, long-chain 3-hydroxyacyl-CoA dehydrogenase deficiency; MCADD, medium-chain acyl-CoA dehydrogenase deficiency; MSUD, maple syrup urine disease; PKU, phenylketonuria; SCD, sickle cell disease; SCID, severe combined immunodeficiency; SMA, 5q spinal muscular atrophy; VLCADD, very-long-chain acyl-CoA dehydrogenase deficiency.

Suppl. Table S3. Participation in the Bavarian Newborn Screening long-term follow-up study (birth cohort 1999-2013).

Condition	Diagnosed	Invited[2]	Participants	Partici-pation-rate	Deceased	Transient	Lost to follow-up	Follow-up ongoing/ completed at age 18	Final or current information available	
	n	n	n	%	n	n	n	n	n	%
Hypothyroidism	480	425	385	90.6	0	51	35	299	350	82.3
CAH[1]	125	121	106	87.6	1[1]	N/A	6	99	100	82.6
Biotinidase deficiency	23	21	20	95.2	0	N/A	6	14	14	66.7
Classic galactosemia	22	22	20	90.9	0	N/A	4	16	16	72.7
PKU	167	160	144	90.0	0	N/A	24	120	120	75.0
MSUD	9	9	9	100	1	N/A	0	8	9	100
MCADD	179	173	164	94.8	2	N/A	13	149	151	87.3
LCHADD	5	5	5	100	2	N/A	0	3	5	100
VLCADD	16	16	15	93.7	0	N/A	2	13	13	81.3
Carnitine cycle defects	6	6	6	100	2	N/A	0	4	6	100
GA I	17	17	16	94.1	0	N/A	0	16	16	94.1
IVA	15	15	13	86.7	0	N/A	2	11	11	73.3
mild HPA	176	0	0	0	0	0	0	0	0	0
**Total**	**1,240**	**990**	**903**	**91.2**	**8**	**51**	**92**	**752**	**811**	**81.9**

[1] One child with an additional diagnosis of MCADD; [2] excluding infants with additional severe or syndromic conditions or malformations

Abbreviations: CAH, congenital adrenal hyperplasia; GA I, glutaric acidemia type I; HPA, hyperphenylalaninemia; IVA, isovaleric acidemia; LCHADD, long-chain 3-hydroxyacyl-CoA dehydrogenase deficiency; MCADD, medium-chain acyl-CoA dehydrogenase deficiency; MSUD, maple syrup urine disease; PKU, phenylketonuria; VLCADD, very-long-chain acyl-CoA dehydrogenase deficiency.

Suppl. Table S4. Reported episodes of fatal metabolic decompensation in participants of the Bavarian NBS long-term follow-up study (birth cohort 1999-2013).

Condition	Age	Context of fatal decompensation
CAH and MCADD	10 months	Severe infection without hospital admission, further details unavailable
MCADD	10 months	Gastrointestinal infection without hospital admission; report of non-adherence to emergency protocols by the medical service called in
MCADD	3 years	Gastrointestinal infection without hospital admission
LCHADD	5 weeks	Septicemia, cardiomyopathy
LCHADD	7 months	Gastrointestinal infection, cardiomyopathy, surgical procedures for placement and revision of PEG (percutaneous endoscopic gastrostomy)
CACT	7 months	Gastrointestinal infection
CPTI	22 months	Details unavailable

Abbreviations: CAH, congenital adrenal hyperplasia; LCHADD, long-chain 3-hydroxyacyl-CoA dehydrogenase deficiency; MCADD, medium-chain acyl-CoA dehydrogenase deficiency.

Suppl. Table S5. Developmental benchmarks achieved by participants of the Bavarian Newborn Screening long-term follow-up study at age six (birth cohort 1999-2013). Note: The developmental benchmarks were achieved by 87.7 % (95 % CI: 86.2-89.1 %) of the children in the reference population [Nennstiel-Ratzel, U.; Lüders, A. et al. Elternfragebögen zu Grenzsteinen der kindlichen Entwicklung im Alter von 1 bis 6 Jahren. Kinderärztliche Praxis 2013, 84, 106–114] [37].

Condition	Individuals	Developmental benchmarks achieved at age six	
	N	n	%
Hypothyroidism	299	276	92.3
CAH	99	95	96.0
PKU	120	109	90.8
MCADD	149	138	92.6
Biotinidase deficiency	14	14	100
Classic galactosemia	16	7	43.8
MSUD	9	6	66.7
LCHADD	3	2	66.7
VLCADD	13	11	84.6
Carnitine cycle defects	4	0	0
GA I	16	13	81.3
IVA	11	10	90.9
**Total**	**753**	**681**	**90.4**

Abbreviations: CAH, congenital adrenal hyperplasia; CI, confidence interval; GA I, glutaric acidemia type I; IVA, isovaleric acidemia; LCHADD, long-chain 3-hydroxyacyl-CoA dehydrogenase deficiency; MCADD, medium-chain acyl-CoA dehydrogenase deficiency; MSUD, maple syrup urine disease; PKU, phenylketonuria; VLCADD, very-long-chain acyl-CoA dehydrogenase deficiency.

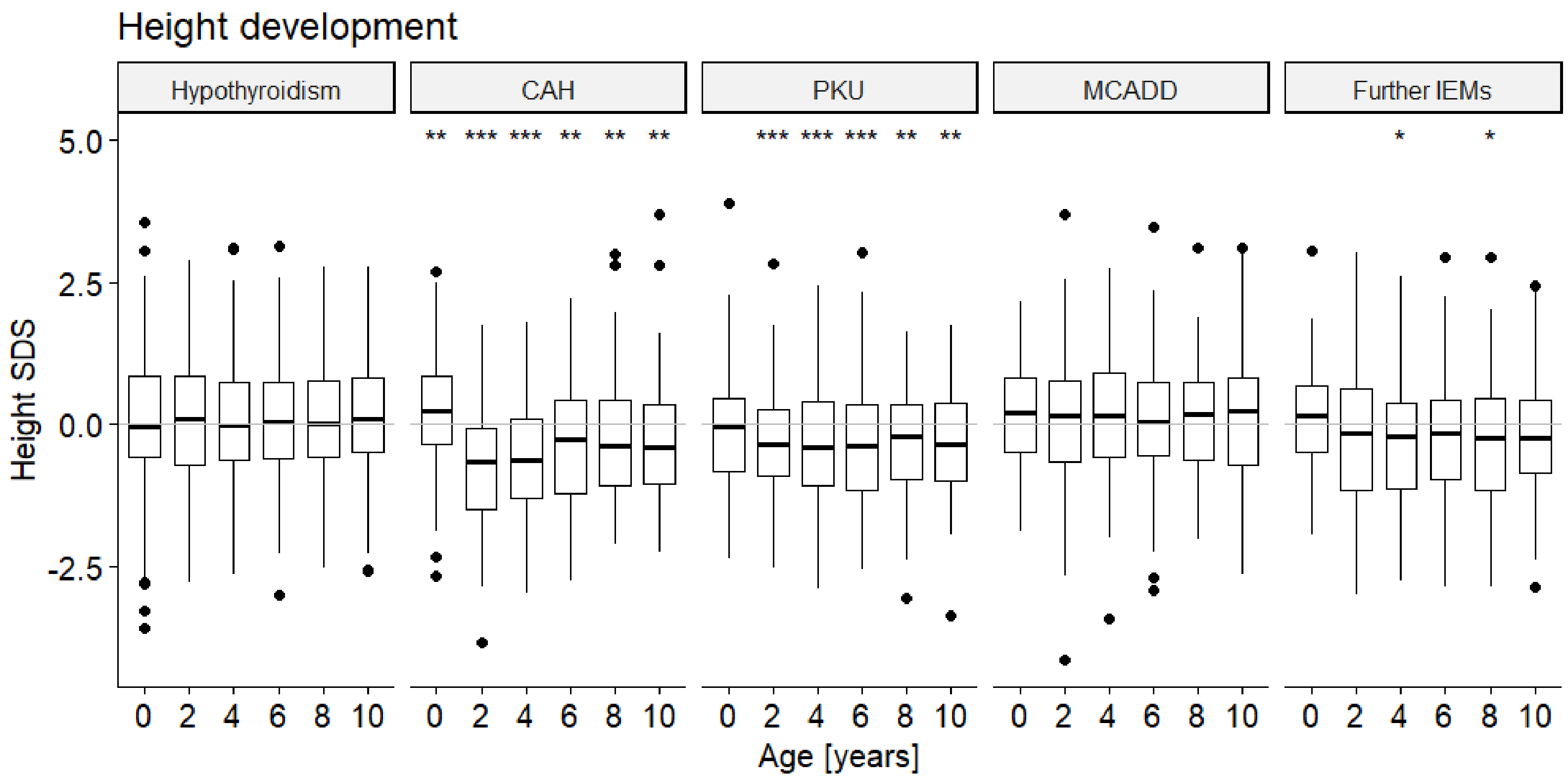

Suppl. Figure S1. Height standard deviation scores (SDS) from birth until age 10 in participants of the Bavarian NBS long-term follow-up study (birth cohort 1999-2013; data available for 773 participants at age 0 and for 662 participants at age 10).
Note: SDS were calculated using the German KiGGS health survey for children and adolescents as a reference [Neuhauser, H.; Schienkiewitz, et al. Referenzperzentile für anthropometrische Maßzahlen und Blutdruck aus der Studie zur Gesundheit von Kindern und Jugendlichen in Deutschland (KiGGS); Beiträge zur Gesundheitsberichterstattung des Bundes, 2011] [33]. Boxes represent the median and IQRs, Whiskers range between the minimum and maximum values, excluding outliers > 1.5 IQR from the box which are represented by small black dots. Significant deviations from the reference population are noted with asterisks: ***, $p < .001$; **, $p < .01$; *, $p < .05$.
Abbreviations: CAH, congenital adrenal hyperplasia; IEMs, inherited errors of metabolism; MCADD, medium-chain acyl-CoA dehydrogenase deficiency; PKU, phenylketonuria; SDS, standard deviation score.

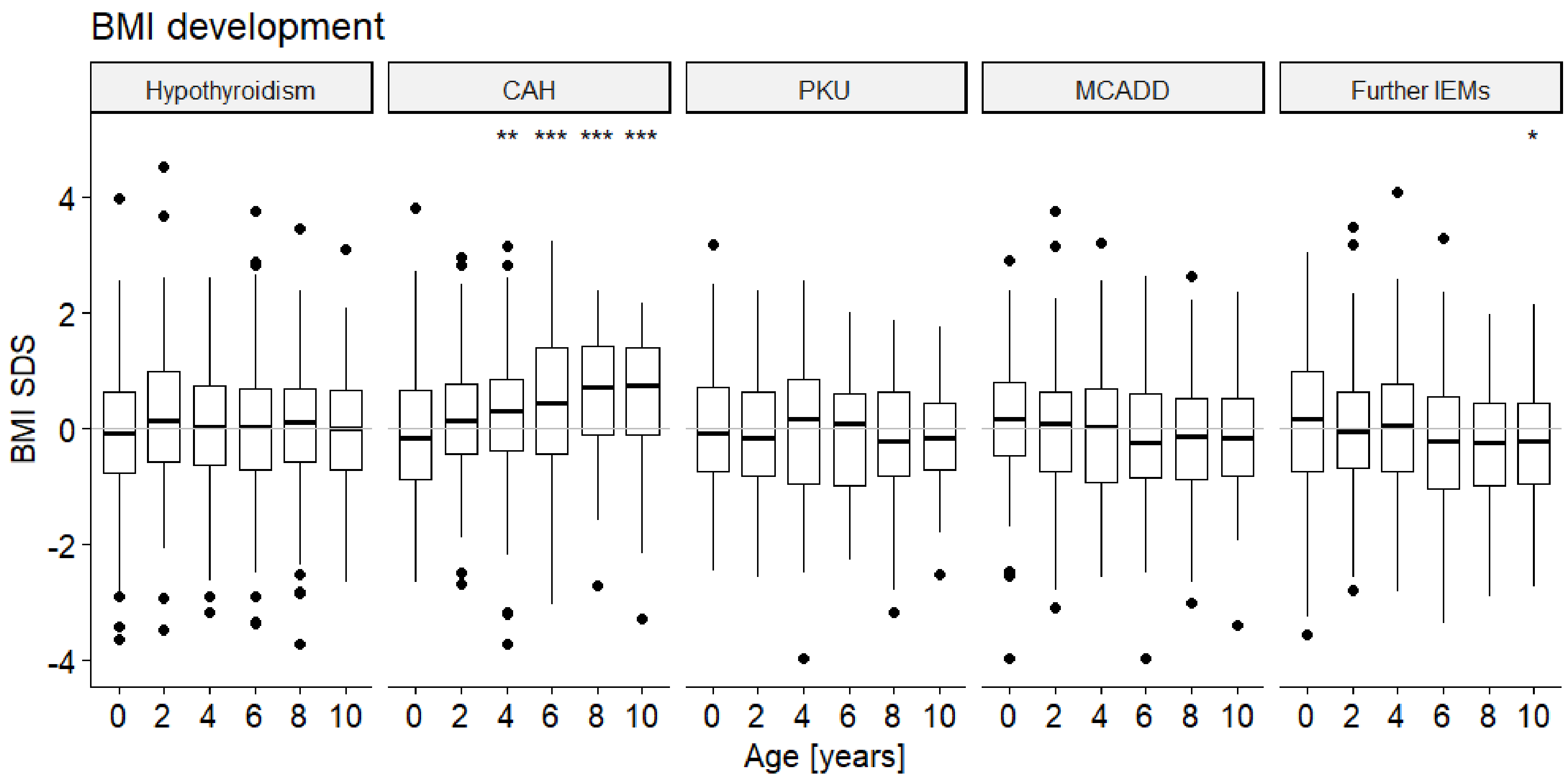

Suppl. Figure S2. BMI standard deviation scores (SDS) from birth until age 10 in participants of the Bavarian Newborn Screening long-term follow-up study (birth cohort 1999-2013; data available for 768 participants at age 0 and for 650 participants at age 10).
Note: SDS were calculated using the German KiGGS health survey for children and adolescents as a reference [Kurth, B.-M.; Schaffrath Rosario, A. Übergewicht und Adipositas bei Kindern und Jugendlichen in Deutschland. Bundesgesundheitsblatt Gesundheitsforschung Gesundheitsschutz 2010, 53, 643–652, doi:10.1007/s00103-010-1083-2.] [34]. Boxes represent the median and IQRs, Whiskers range between the minimum and maximum values, excluding outliers > 1.5 IQR from the box which are represented by small black dots. Significant deviations from the reference population are noted with asterisks: ***, $p < .001$; **, $p < .01$; *, $p < .05$.
Abbreviations: CAH, congenital adrenal hyperplasia; IEMs, inherited errors of metabolism; MCADD, medium-chain acyl-CoA dehydrogenase deficiency; PKU, phenylketonuria; SDS, standard deviation score.

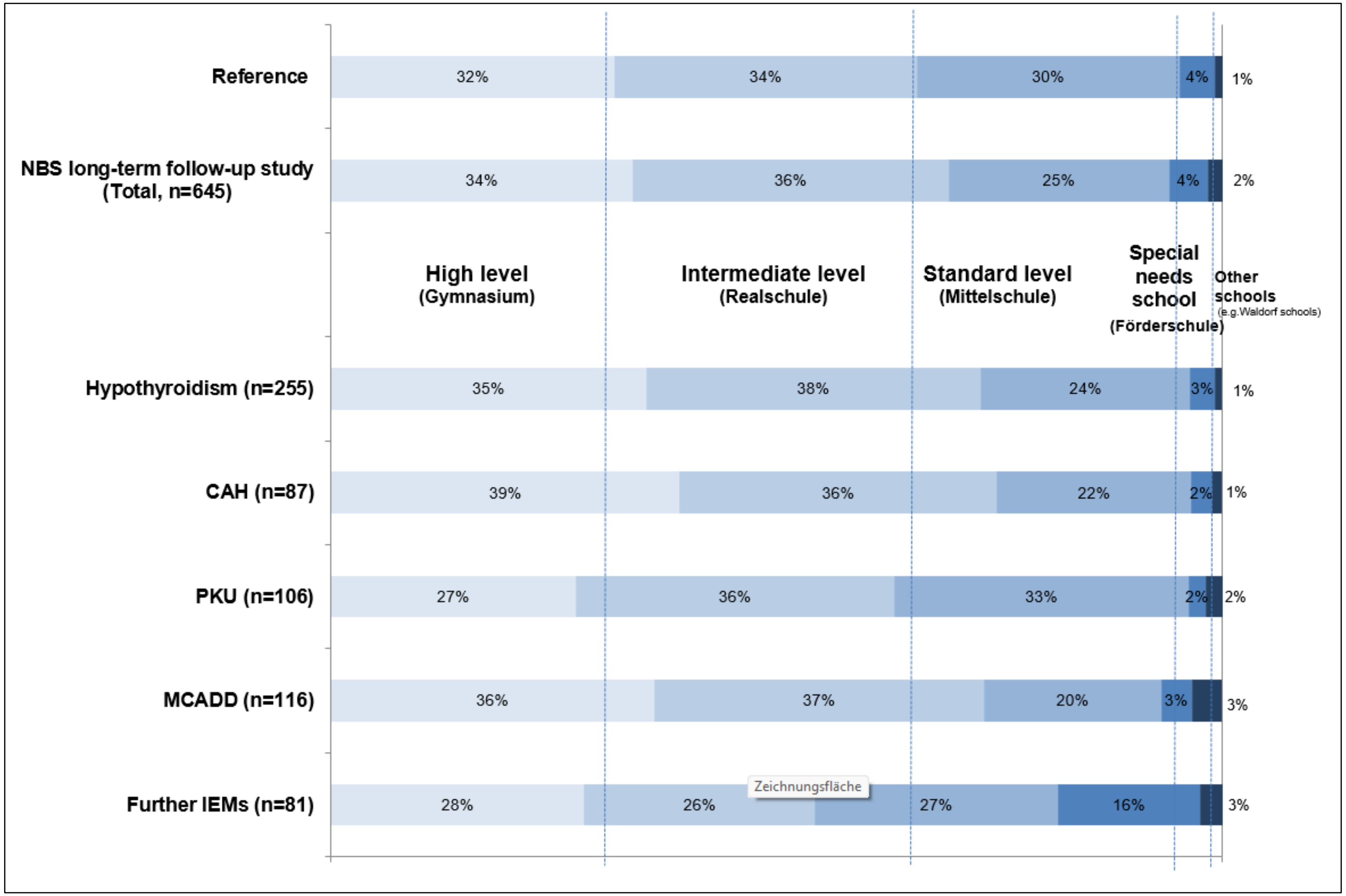

Suppl. Figure S3. School careers in participants of the Bavarian Newborn Screening long-term follow-up study at age 14 (birth cohort 1999-2013, data available for 645 participants) compared to the Bavarian reference population.

Reference: Bavarian State statistics on the distribution of pupils in grade 8 from 2013/2014 to 2022/23 [Bayerisches Staatsministerium für Unterricht und Kultus. Bayerns Schulen in Zahlen 2022/2023. Schriften des Bayerischen Staatsministeriums für Unterricht und Kultus Reihe A Bildungsstatistik 2023, p. 19] [38].

Note: Bavaria's secondary school system is divided into different levels based on pupils' school performance.

Abbreviations: CAH, congenital adrenal hyperplasia; IEM, inherited error of metabolism; MCADD, medium-chain acyl-CoA dehydrogenase deficiency; PKU, phenylketonuria.

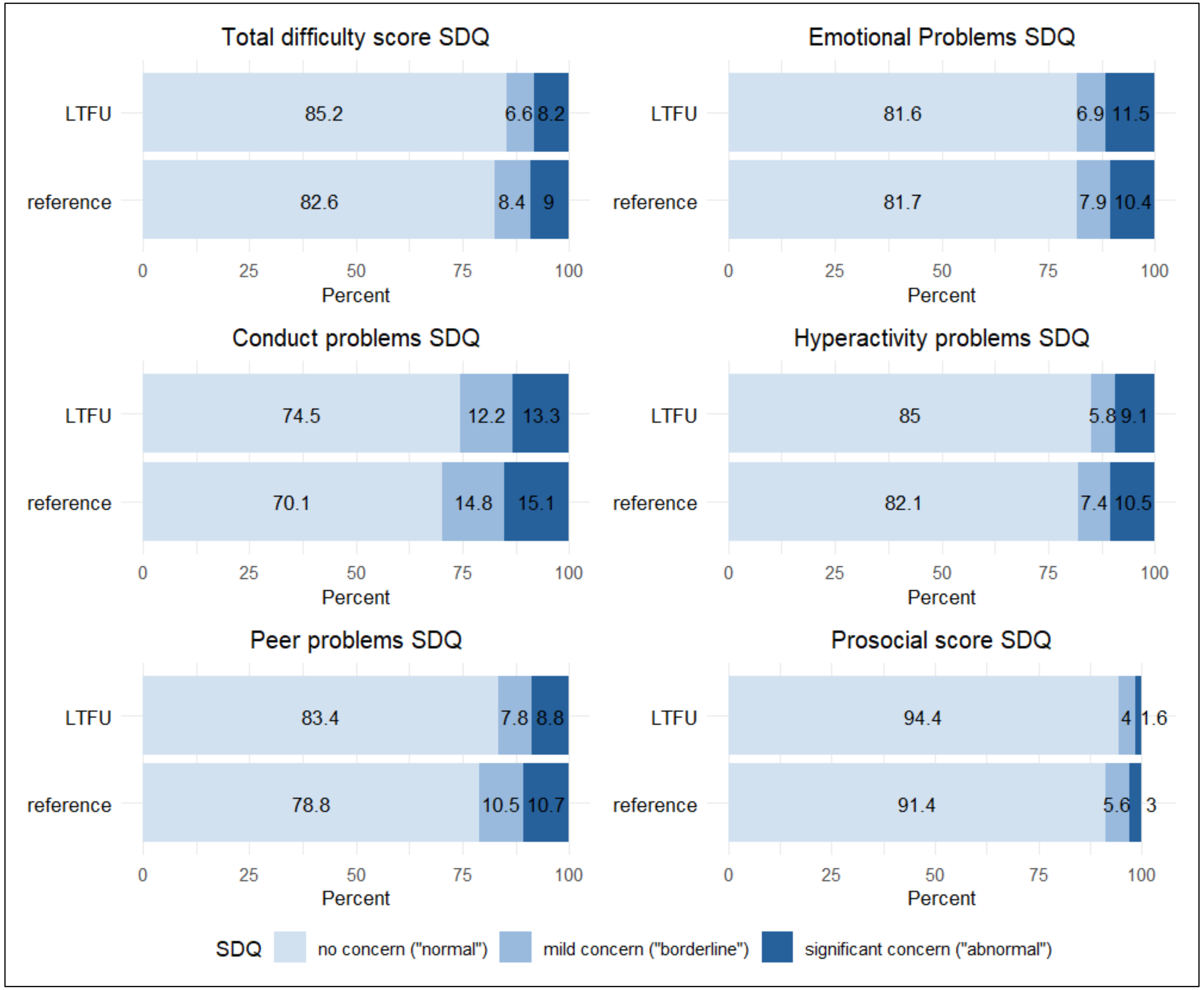

Suppl. Figure S4. SDQ results in participants of the Bavarian NBS long-term follow-up study at age 8 (birth cohort 1999-2013; data available for 547 participants).
Reference: German KiGGS health survey for children and adolescents [Hölling, H.; Erhart, M. et al. Verhaltensauffälligkeiten bei Kindern und Jugendlichen. Erste Ergebnisse aus dem Kinder- und Jugendgesundheitssurvey (KiGGS). Bundesgesundheitsblatt Gesundheitsforschung Gesundheitsschutz 2007, 50, 784–793, doi:10.1007/s00103-007-0241-7] [41]. **Abbreviations:** SDQ, Strengths and Difficulties Questionnaire; LTFU, long-term follow-up study.
